# Physiological Characterization of Vestibular Efferent Brainstem Neurons Using a Transgenic Mouse Model

**DOI:** 10.1371/journal.pone.0098277

**Published:** 2014-05-27

**Authors:** Sara Leijon, Anna K. Magnusson

**Affiliations:** 1 Center for Hearing and Communication Research, Karolinska Institutet, Stockholm, Sweden; 2 Department of Clinical Science, Intervention and Technology, Unit of Audiology, Karolinska University Hospital, Stockholm, Sweden; Universitat Pompeu Fabra, Spain

## Abstract

The functional role of efferent innervation of the vestibular end-organs in the inner ear remains elusive. This study provides the first physiological characterization of the cholinergic vestibular efferent (VE) neurons in the brainstem by utilizing a transgenic mouse model, expressing eGFP under a choline-acetyltransferase (ChAT)-locus spanning promoter in combination with targeted patch clamp recordings. The intrinsic electrical properties of the eGFP-positive VE neurons were compared to the properties of the lateral olivocochlear (LOC) brainstem neurons, which gives rise to efferent innervation of the cochlea. Both VE and the LOC neurons were marked by their negative resting membrane potential <−75 mV and their passive responses in the hyperpolarizing range. In contrast, the response properties of VE and LOC neurons differed significantly in the depolarizing range. When injected with positive currents, VE neurons fired action potentials faithfully to the onset of depolarization followed by sparse firing with long inter-spike intervals. This response gave rise to a low response gain. The LOC neurons, conversely, responded with a characteristic delayed tonic firing upon depolarizing stimuli, giving rise to higher response gain than the VE neurons. Depolarization triggered large TEA insensitive outward currents with fast inactivation kinetics, indicating A-type potassium currents, in both the inner ear-projecting neuronal types. Immunohistochemistry confirmed expression of Kv4.3 and 4.2 ion channel subunits in both the VE and LOC neurons. The difference in spiking responses to depolarization is related to a two-fold impact of these transient outward currents on somatic integration in the LOC neurons compared to in VE neurons. It is speculated that the physiological properties of the VE neurons might be compatible with a wide-spread control over motion and gravity sensation in the inner ear, providing likewise feed-back amplification of abrupt and strong phasic signals from the semi-circular canals and of tonic signals from the gravito-sensitive macular organs.

## Introduction

Our hearing and balance sensory organs in the inner ear do not only convey information about sound and motion from the periphery to the brain. The brain also exerts powerful control over the cochlea and the vestibular apparatus through efferent neurons in the lower brainstem. In mammals, the olivocochlear efferent system has been widely investigated and demonstrated to generate a feed-back loop that adjusts the sensitivity of hearing in a frequency-dependent manner [16]. The vestibular system has a homologous efferent innervation of the motion and gravity sensors. These vestibular efferent nerve fibers are heavily outnumbered by the afferent fibers, despite ample efferent synaptic contacts with the vestibular epithelia [25], [26]. This has been shown to depend on the rich arborization of vestibular efferents, which can make multiple synaptic contacts with afferent nerve endings and hair cells in the vestibular sensory epithelia [5], [23]. The vestibular efferent (VE) neurons provide presynaptic innervation of the type II hair cells, which are primarily inhibited [6], [12], [29], and postsynaptic innervation of afferent nerve-endings of both type I and II hair cells, which are predominantly excited [5], [25], [45]. In addition, each efferent fiber may innervate more than one vestibular sensory end-organ, and thereby, exert control over both the semicircular canal and the macular organs [25], [26].

The functional role of this widespread vestibular efferent projection has been elusive. One main hypothesis has been that the vestibular efferent system may quench the afferent vestibular response during self-generated motion. This notion was based upon observations that the sensitivity of second order vestibular neurons in the primate brainstem decreases during self-generated movements [44], [56]. Research in the toadfish provided a possible mechanism by demonstrating that behaviorally-induced excitation of the vestibular efferents reduces the rotational sensitivity of the vestibular primary afferents [5], [6], [27]. However, when comparing the sensitivity of semicircular canal afferents during active and passive head-movements in awake behaving monkeys, no difference was found [17], indicating that vestibular efference must have another role.

To understand how the vestibular efferents affect the signal processing in the vestibular sensory epithelia and afferents, it is essential to investigate the properties of the VE neurons themselves. The VE neurons comprise a small group of cholinergic cells located in the dorsal brainstem [11], [24], [25], [46]. Physiological investigations of VE neurons have hitherto been hampered by their small number, which precludes identification based on cytoarchitectural borders. In order to make it possible to single out VE neurons in a brain slice preparation, a transgenic mouse expressing eGFP in cholinergic neurons under a broad choline-acetyltransferase (ChAT)-spanning locus was explored in this study [66]. Specific expression of eGFP in VE neurons enabled the first physiological characterization of these neurons by using eGFP guided patch-clamp recordings. By comparing the electrical properties of VE neurons to the well-investigated lateral olivocochlear neurons [2], [22], [62], we find selective properties of VE neurons that may shed light on their role for vestibular sensation.

## Methods

### Animal model

In this study, mice of C57BL/6 background were bred with a bacterial artificial chromosome (BAC) eGFP-ChAT mouse, in which eGFP was knocked-in under a cholinergic locus which contains the ChAT and the vesicular acetylcholine transporter (VAChT) genes [66].

For *in vitro* slice preparation, mice of postnatal day (P) 6 to 18 (VE: P7–P18; LOC: P6–16) were used and the eGFP expression was confirmed in the brain slice with a fluorescent microscope with the appropriate filter settings. Care was taken to minimize exposure to fluorescent illumination. For immunohistochemistry, adult BAC-eGFP and wildtype mice of the C57BL/6 strain were used. In 3 cases, wildtype Sprague Dawley rats were used. In order to identify eGFP positive individuals, the mice were genotyped with respect to the eGFP insertion using primers from ChAT (chat7220-22 bp; agtaaggctatgggattcattc) and from eGFP (G20 bp-496; agttcaccttgatgccgttc), respectively.

The animals were decapitated (for brain slices) or perfused (for anatomical preparation) following an overdose of sodium-pentobarbital. All experiments were performed in conformity with the rules set by the European Commission Council Directive (86/89/ECC) and approved by the local Swedish Animal Care and Use Committee (Dnr N32/07 and N13/10).

### Electrophysiology

Whole-cell current and voltage clamp recordings were made at room temperature using a Multiclamp 700B amplifier (Molecular Devices, U.S.A) as described previously [20]. Briefly, borosilicate glass microelectrodes (GC150F-10, Harvard Apparatus, UK) were pulled on a vertical electrode puller (PP-830, Narashige, Japan) yielding a final tip resistance of 5–9 MΩ. The internal pipette solution contained (in mM): 130 K-gluconate, 5 KCl, 10 HEPES, 1 EGTA, 2 Na_2_-ATP, 2 Mg-ATP, 0.3 Na_3_-GTP, 10 Na_2_-phosphocreatinine, adjusted to pH 7.3 with KOH.

The bridge balance was applied for current clamp recordings. For voltage clamp recordings, the series resistance was compensated by 70–80% and monitored throughout the experiment. The signals were filtered with a low-pass 4-pole Bessel filter at 10 kHz, sampled at 20 kHz and digitized using a Digidata 1440A interface (Molecular Devices, U.S.A.). Stimulus generation, data acquisition and off-line analysis of data were performed using the pClamp Software (Version 10.0, Molecular Devices; U.S.A.). The voltages have been corrected for a junction potential of −11.6 mV. The input resistance and the membrane time constant were estimated from the voltage responses to small current injections (±20 pA) around rest by, respectively, calculating the slope of current-voltage plot or fitting a single exponential function. The electrophysiological results are expressed as mean ± standard deviation (S.D.) in the text and as mean ± standard error of the mean (S.E.M.) in the figures. The level of significance was determined by Student's unpaired t-test (p<0.05 was considered statistically significant). All drugs were bath applied as follows: 0.5 µM tetrodotoxin (TTX; Tocris) was used to block sodium currents and 500 µM tetraethylammonium chloride (TEA; Sigma) and 400 µM 4-aminopyridine (4-AP; Sigma) were used to block potassium currents.

### Preparation of tissue for immunohistochemistry

Two different techniques were used for tissue fixation and sectioning. In the first one, transcardial perfusion, the animal was euthanized with an overdose of sodium-pentobarbital. An initial 50 mL 0.9% NaCl is followed by approximately 200 mL of ice-cold 4% PFA in 0.1 M phosphate buffered saline (PBS). The dissected brain was post-fixed for 2–3 h in 4% PFA at 4°C and then cryoprotected at 4°C overnight in a solution of 30% sucrose in 0.1 M PBS. A Leica CM3050 S (Leica Microsystems, Germany) cryostat was used for sectioning of the brain in 30–50 µm thick transverse sections. Sections were collected in multi-well plates filled with PBS.

In the second technique, the shock-freeze method, the animal was similarly euthanized by an overdose of sodium-pentobarbital, whereafter it was decapitated and the brain carefully dissected, covered with Tissue-Tek (Sakura Finetek, Netherlands) and immediately frozen in isopentane and dry-ice. Until sectioning, the samples were kept at −20°C. The unfixed tissue was sectioned with the same cryostat in 12 µm thick transverse sections that were mounted directly onto SuperFrost glass slides (Menzel-Gläser, Germany). After about 30 min at room temperature, the tissue was fixed for 20 min in 4% PFA in 0.1 M PBS and washed three times with PBS, after which the staining was started immediately.

### Immunohistochemistry

Initially sections are pre-incubated in 5–10% normal donkey serum in a blocking solution (BS) of 1% bovine albumin serum (BSA) and 0.3% Triton X-100 in PBS for 1 h at room temperature. Next, sections are incubated with the primary antibodies overnight at 4°C in BS containing 2% normal donkey serum. Primary antibodies used were AlexaFluor488-conjugated rabbit anti-GFP 1∶500 (Invitrogen, Corp., Carlsbad, CA), goat anti-ChAT 1∶100 (Millipore, Corp., Temecula, CA), rabbit anti-Kv4.2 1∶100 and rabbit anti-Kv4.3 1∶100 (Alomone Labs, Ltd., Jerusalem, Israel). After three washes with PBS, sections are incubated in darkness with Cy3-conjugated donkey anti-goat and/or Cy2-conjugated donkey anti-rabbit (Dianova, GmbH., Hamburg, Germany) in BS for 2 h at room temperature. Sections are washed with PBS, gelatin coated and cover-slipped with an anti-fading medium and kept in the dark at −20°C until visualization. The specificity of the immunoreactions was confirmed by either omitting the primary antibody, or by adding the corresponding immunopeptide in excess at the step of primary antibody incubation (data not shown). A summary of all antibodies used in this study is provided in Table 1.

**Table 1:** Primary antibodies used.

Antibody	Target sequence	Source	Dilution
goat α-ChAT	ChAT isoform 2	Chemicon (Millipore) Cat no. AB1440	1∶200–1∶100
rabbit α-GFP-AlexaFluor488		Invitrogen Cat no. A-21311	1∶500
rabbit α-Kv4.2	Intracellular C-terminus of rat K_v_4.2	Alomone Cat no. APC-023	1∶400–1∶100
rabbit α-Kv4.3	Intracellular C-terminus of human K_v_4.3	Alomone Cat no. APC-017	1∶400–1∶100

### Visualization of immunoreactivity

Immunolabeling was visualized with light microscopy using a Zeiss Observer Z1 fluorescence microscope (Carl Zeiss, Germany) equipped with a Zeiss AxioCam MRm camera and digitally processed using AxioVision 4.8. Confocal optical sections were acquired with a Zeiss LSM 510 confocal laser-scanning microscope (Carl Zeiss, Germany) equipped with Plan-Apochromat 63×/1.4 and 100×/1.4 DIC oil immersion objectives. Fluorochromes were visualized using an argon laser with excitation wavelengths of 488 nm (peak emission 509 nm) for GFP and a He-Ne laser with a laser line of 543 nm (peak emission 570 nm) for Cy3. For each optical section, the images were collected sequentially for the two fluorochromes. Stacks of eight-bit grayscale images were obtained with axial distances of 100 nm between optical sections and pixel sizes of 20–200 nm depending on the selected zoom factor (0.7–7). After stack acquisition, Z chromatic shift between color channels was corrected. RGB stacks, montages of RGB optical sections, and maximum-intensity projections were created using AxioVision 4.8. In order to de-noise images, stacks of light optical sections were deconvolved with the ImageJ plugin for parallel iterative deconvolution 3D (method: WPL algorithm; boundary: reflexive; max iterations: 5; max number of threads (power of 2∶2) using theoretical point-spread functions (PSF).

## Results

### Co-localization of ChAT and GFP in vestibular efferents and motoneurons

**Figure 1:**
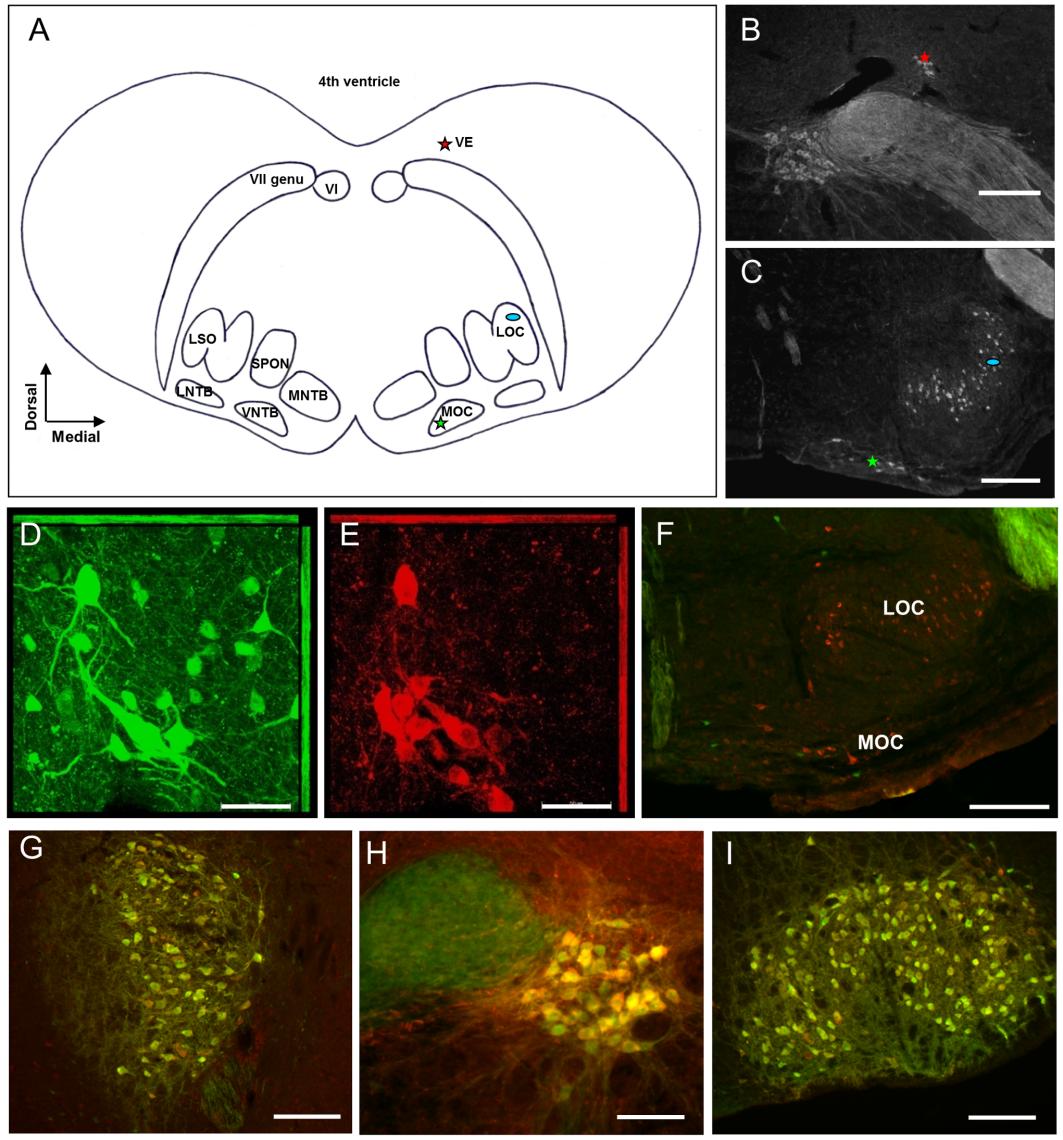
VE neurons are cholinergic and express eGFP. (A) Schematic view of transversely sectioned mouse auditory brainstem. The left side delineates the nuclei of interest and adjacent landmarks whereas the right side marks the localization of the inner-ear projecting neurons. The vestibular efferents (VE, red star) reside dorsolaterally to the VII nerve genu, and the lateral (LOC, blue oval) and medial (MOC, green star) olivocochlear efferents are localized in and around the lateral superior olive (LSO) and the ventral nucleus of the trapezoid body (VNTB), respectively. (B–C) Corresponding immunofluorescent ChAT-labeling used to localize the inner ear-projecting neurons in the brainstem. (D–E) Confocal laser scanning microscopy images of adult auditory brainstem co-labeled with anti-GFP (green) and anti-ChAT (red) demonstrate that the cholinergic VE neurons express eGFP in the ChAT-mouse. (F) Co-labeling of anti-GFP (green) and anti-ChAT (red) in the LSO and the VNTB demonstrate that cholinergic LOC and MOC neurons are lacking eGFP-expression. A few non-cholinergic cells displayed ectopic expression of eGFP. (G–I) Motoneurons in the nuclei of the trigeminal nerve (N.V), the abducens nerve (N.VI) and the facial nerve (N.VII), in order from left to right, are strongly co-labeled for GFP and ChAT. Scale bars: (B–C; F–G; I) 200 µm, (H) 100 µm and (D–E) 50 µm.

The VE neurons reside in the dorsal brainstem close to the fourth ventricle, where they form a small group of neurons dorsolaterally to the genu of the facial nerve [3], [7], [24] whereas the olivocochlear efferents are located in in the ventral brainstem in the superior olivary complex [1], [10]; (Fig. 1A). In order to identify the VE neurons in the brainstem based on their location, immunohistochemistry against the ChAT protein was first performed in wild-type mice. The VE cell group was distinctly located in proximity to the facial nerve genu and the abducens nucleus (Fig. 1A and B). Likewise, the cholinergic olivocochlear efferents were confirmed in the superior olivary complex (Fig. 1A and C). Next, we performed double immunohistochemistry against eGFP and ChAT to verify the specificity of the eGFP expression in VE and olivocochlear neurons in mice genotyped positive for the eGFP insertion. This resulted in robust co-labeling of eGFP and ChAT in the VE neurons (Fig. 1D and E). However, no overlap between eGFP and ChAT immunolabeling was found in the olivocochlear efferent neurons (Fig. 1F). Neither the LOC neurons in the lateral superior olive nor the medial olivocochlear (MOC) neurons in the ventral nucleus of the trapezoid body displayed eGFP immunoreactivity (Fig. 1F). Furthermore, sporadic ectopic expression of eGFP in non-cholinergic neurons was observed in vicinity of the areas of interest (Fig. 1F). In contrast to the lack of eGFP expression in the cholinergic olivochlear efferents, robust double immunolabeling of eGFP and ChAT was observed in nearby brainstem motoneurons, such as in the trigeminal (Fig. 1G), the abducens (Fig. 1H) and the facial nucleus (Fig. 1I). The distribution of eGFP immunoreactivity was not age-dependent as the same eGFP pattern was found in brain slices from the pups used for electrophysiology.

Although the olivocochlear efferent neurons were not highlighted in this eGFP-ChAT mouse, the selective expression of eGFP in the VE area enabled, for the first time, targeted intracellular electrophysiological recordings from VE neurons in a brain slice by utilizing a fluorescent microscope in combination with a patch clamp amplifier. For comparison, patch-clamp recordings were also obtained from LOC neurons, which are easily located in the lateral superior olive and identified based on their characteristic physiology [22], [62] and morphology [2]. The MOC efferent neurons were excluded from investigation since they are more heterogeneously distributed [9], [10], which precludes unambiguous identification. In addition, the vestibular epithelia lack the specialized outer hair cells of the cochlea [54], thus further motivating exclusion of the MOC neurons for the time being.

### Comparison of basic membrane properties between VE and LOC neurons

**Figure 2:**
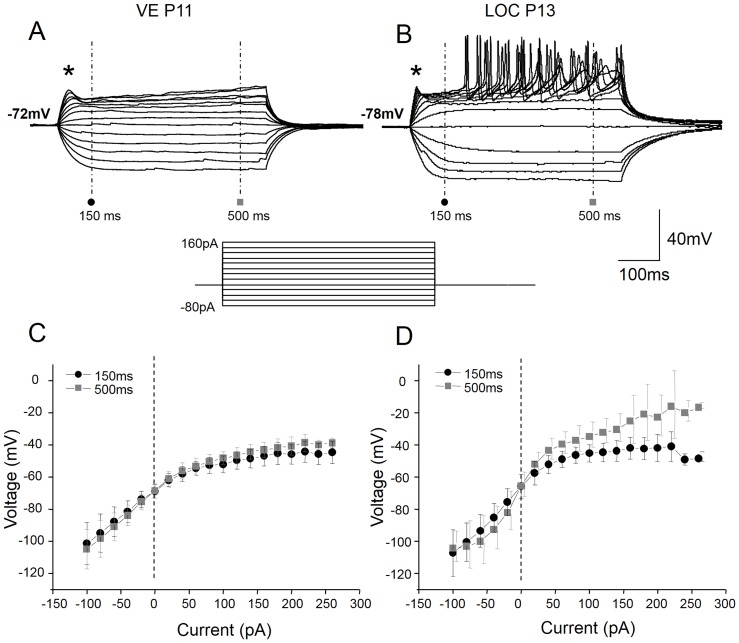
VE neurons have non-linear voltage properties in the depolarizing range. Voltage responses of VE (A) and LOC (B) neurons to depolarizing current steps of 20 pA (current protocol displayed below the voltage traces) injected from the resting membrane potential. The VE neurons responded with a subtle depolarizing shoulder (asterisk), which failed to trigger action potentials, whilst the LOC neurons show a more pronounced depolarizing shoulder, larger voltage deflections to the same current increments and delayed firing of action potentials upon positive current injection. The voltage-current relationship measured, with respect to the beginning of the recording, both at the onset (150 ms; black circles) and at steady-state (500 ms; grey squares) of the response, displayed strong outward rectification (a decreased slope) in the depolarizing range in the VE neurons, whereas it was near linear in the hyperpolarizing range (C). The LOC neurons also displayed a voltage-dependent non-linearity at the onset of depolarization, but were more linear at steady state voltages (D). The larger variance at steady state are due to the contamination of the spiking activity at the higher current levels in the LOC neurons.

Whole-cell patch-clamp recordings were obtained from nine eGFP-positive VE neurons and from thirteen physiologically identified LOC neurons in the lateral superior olive. First, the basic membrane properties of VE neurons were characterized and compared with those of LOC neurons (Table 2). The VE neurons, identified by their eGFP expression, had hyperpolarized membrane potentials (−76.5±2.7 mV). When injected with negative currents, VE neurons consistently lacked a sag in their voltage response (Fig. 2A). These features are very similar to those described for LOC neurons in the rat [2], [22]. Indeed, recordings from LOC neurons, identified based on their location in the lateral superior olive and their small soma size, displayed equally low resting membrane potential (−75.7±1.9 mV; p = 0.8) and complete absence of a voltage sag upon hyperpolarization (Fig. 2B). These two membrane properties are indicative of little to no hyperpolarization activated current, Ih [51]. In line with this notion, both VE and LOC neurons displayed qualitatively similar linear voltage-current relationships in the hyperpolarizing range (Fig. 2C and D) even though VE neurons had smaller voltage deflections. When injected with small (20 pA) positive currents, the VE neurons displayed a smooth depolarizing shoulder (marked by the asterisk in Fig. 2A). Furthermore, the voltage-current relationship was non-linear at both the initial (150 ms from the start of the recording) and at steady state (500 ms from the start of the recording) depolarization (Fig. 2A and C). The LOC neurons displayed larger voltage deflections to the same current increments. As a result of this, they reached spike threshold and fired action potentials (Fig. 2B). Nevertheless, the same current protocol was used for comparison of other basic properties. The LOC neurons also displayed a depolarizing shoulder, which became sharper with increasing current levels (Fig. 2B). However, although LOC neurons fired plenty of action potentials, the first depolarizing shoulder never reached threshold (Fig. 2B). Their initial (150 ms) voltage-current relationship followed a similar non-linear trend as observed in VE neurons (Fig. 2D). The more linear voltage-current relationship and the larger variance at steady state (500 ms) reflect contamination of spiking activity. Taken together, both VE and LOC neurons appear to have transient depolarization-activated currents that suppress action potentials. In addition, VE neurons seem to have more sustained depolarization-activated currents than LOC neurons.

**Table 2:** Summary of basic membrane properties.

	VE	LOC	*P*
RMP, mV	−76.49±2.68	−75.68±1.90	0.801
Rin (+20 pA), Mohm	387.2±52.0	848.0±84.6	0.001**
Rin (−20 pA), Mohm	541.2±165.3	870.9±80.8	0.058
Tau, ms	30.66±4.06	46.17±8.73	0.022*
Capacitance, pF	30.6±5.4	15.4±2.1	0.007**
AHP, mV	20.56±2.69	13.38±1.23	0.014*
AP peak, mV	69.89±3.77	74.85±2.73	0.287
AP half-width, ms	2.07±0.22	1.54±0.08	0.016*
AP rise, mV/ms	104.4±11.4	160.2±16.3	0.016*
Decay time, ms	2.41±0.43	1.36±0.07	0.009**
AP threshold, mV	−46.71±2.72	−46.91±1.25	0.943
Max frequency, Hz	7.43±1.29	11.55±3.66	0.020*

*p<0.05,

** p<0.01 by Students unpaired t-test.

In order to investigate the membrane properties in the de- and hyperpolarizing range, the smallest sub-threshold current step (20 pA) was used to estimate the input resistance and membrane time constant in VE and LOC neurons, respectively. In accordance with smaller voltage deflections triggered by small depolarizing currents, VE neurons have a significantly lower input resistance in the depolarizing range than LOC neurons (VE: 387±201 Mohm; LOC: 848±99 Mohm, p = 0.001). The average input resistance in the hyperpolarizing range was also smaller in VE neurons than in LOC neurons and almost reached statistical significance (VE: 541±181 Mohm; LOC: 871±81 Mohm, p = 0.058). To get an estimate of the integrative property, the membrane time constant was derived from fitting a single exponential function to the decline of the depolarizing current step. This revealed a significantly faster membrane time constant in VE neurons (VE: 31±4.1 ms; LOC: 46±8.7 ms, p = 0.002) than in LOC neurons. Since the membrane time constant is dependent on the cell size, the cell capacitance was estimated from the read-out on the amplifier (Table 2). The compensating capacitance was 2-fold higher for VE than for LOC neurons (VE: 31±5 pF; LOC: 15±2 pF; p = 0.007). This indicates that the cell size and morphology may be an important factor in influencing the functional role of each inner ear-projecting neuronal population.

### VE neurons have distinct action potential and response properties from LOC neurons

**Figure 3:**
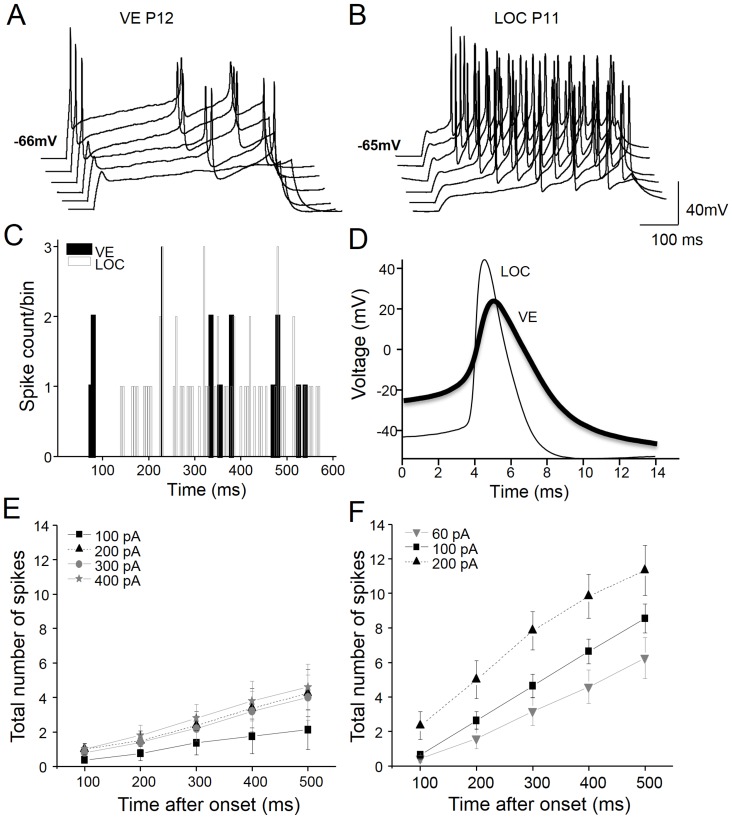
Distinct firing patterns of VE and LOC neurons. Representative examples of spiking discharge to depolarizing current steps of 100(A) and 20 pA in a LOC neuron (B). Note that a small offset has been introduced between the voltage traces to avoid overlap of the spikes. Peri-stimulus time histogram with a bin size of 5 ms corresponding to the spiking activity in the VE (black bars) and the LOC (grey bars) traces displayed above (C). The first action potential in response to current injection (rheobase) in the voltage traces from the examples above (D). VE neurons characteristically fire action potentials at the onset of the stimulus followed by long inter-spike intervals that result in sparse firing (A, C). The LOC neurons, conversely, display long first spike latencies, after which the neuron fires throughout the stimulus (B, C). The mean input-output curves (firing rate during the first 500 ms of the stimulus as a function of input current) were linear and gave rise to more shallow response gains for the VE, n = 8 (E) than for the LOC, n = 12 (F) neurons.

Upon depolarization, VE neurons consistently fired a single spike (onset spiking) followed by a long inter-spike interval (ISI), resulting in sparse firing (Fig. 3A and C). In contrast to the robust onset spiking observed in VE neurons, LOC neurons where characterized by a long first spike latency at rheobase, followed by a tonic firing pattern (Fig. 3B and C). Thus, VE and LOC neurons responded complementary to supra-threshold depolarizing stimuli. The VE neurons marked the onset whereas the LOC neurons highlighted the duration of the depolarization (Fig. 3C).

**Figure 4:**
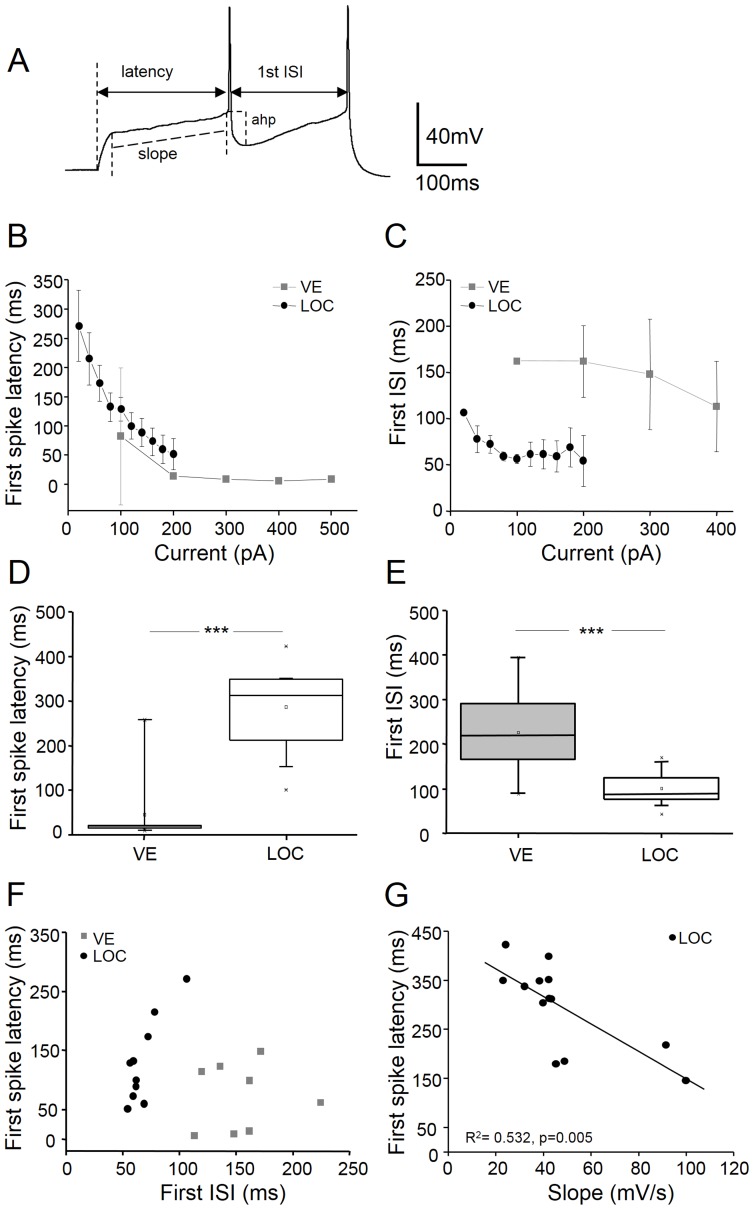
First spike latency and discharge regularity characterize VE and LOC neurons. An example voltage trace (A) illustrating how the following parameters were measured: *latency*: from the stimulus onset to the threshold of the first action potential; *first inter-spike interval (ISI)*: from the peak of the first spike to the peak of the following spike; *afterhyperpolarization (ahp)*: from the spike threshold to the deepest voltage of spike repolarization; *slope*: a linear fit was made to the voltage slope between the depolarizing shoulder leading up to the threshold of the first spike. The first spike latency (B) and the first ISI (C) plotted against current strength in VE (grey squares) and LOC (back circles). The mean values of the first spike latency (D) and the first ISI (E) for VE and LOC neurons at rheobase current levels (VE rheobase: 180±50 pA; LOC rheobase: 44±4 pA). Scatter plot of the mean values of the first spike latencies versus the first ISIs demonstrate that VE and LOC neurons cluster separately (F). The first spike latency is negatively correlated (R^2^ = 0.534, p = 0.005; Pearson's correlation) against the voltage slope leading up to the first spike in LOC neurons (G). *** p<0.001 by Students unpaired t-test.

One factor that may govern the firing pattern is the shape and kinetics of the action potential. Somewhat surprisingly, although VE neurons have a faster membrane time constant than LOC neurons, this was not reflected in the kinetics of their action potentials (Fig. 3D). VE neurons had a 35% slower action potential rise (VE: 104±11 mV/ms; LOC: 160±16 mV/ms; p = 0.016) and a 71% slower decay time (VE: 2.4±0.4 ms; LOC: 1.4±0.1 ms; p = 0.009), which resulted in a 34% longer action potential half-width (VE: 2.07±0.22 ms; LOC: 1.54±0.08 ms; p = 0.016) (Fig. 3D; Table 2,). Moreover, the action potentials of VE neurons were not just slower and wider, they also displayed a deeper after-hyperpolarization, measured from spike threshold to the deepest voltage deflection following spiking (AHP; Fig. 4A) than LOC neurons (VE: 20.5±2.69 mV; LOC: 13.38±1.23 ms; p = 0.014; Table 2). However, the mean action potential threshold and spike amplitude did not differ between VE and LOC neurons (Table 2).

Another important property that reflects the neuron functionality is their responsiveness to stimulus strength. Therefore, the input-output functions (firing vs. the stimulus duration for different current strengths) were estimated for both types of neurons. The VE neurons were characterized by sparse firing, yielding shallow input-output functions (Fig. 3E). When stimulated with 300 pA current steps, VE neurons responded with a maximal firing frequency of 7.4±1.2 Hz at steady state stimulation (last 300 ms of the stimulus), and the spiking gave rise to a coefficient of variation (CV) of 1.8. The LOC neurons were more responsive to the stimulus strength and, as a consequence they displayed steeper input-output functions (Fig. 3F) and reached a higher maximal firing rate (11.6±1.0 Hz, p = 0.02) compared to VE neurons. Also, the CV of firing during steady state stimulation was lower, 0.8 in the LOC neurons, indicating that LOC neurons had less variance in their response to long-lasting depolarizations than VE neurons.

### VE and LOC neurons can be categorized based on their spike latency and inter-spike interval

A hallmark of VE neurons was their onset response and sparse firing during prolonged stimulation whereas LOC neurons where characterized by their long first spike latency. This result called for closer inspection of the first spike latency, measured from the stimulus onset to the first spike threshold (Fig. 4A), and the first ISI, measured from the peak of the first spike to the peak of the second spike (Fig. 4A). In VE neurons, the first spike latency decreased over two-fold between 100 and 200 pA current steps, after which it remained constant at values around 10 ms for larger current strengths (Fig. 4B). In LOC neurons, the first spike latency declined steeply as a function of the current level in a monotonic fashion (Fig. 4B). On average, the first spike latency measured at rheobase was more than six times longer in LOC neurons (286±17 ms; rheobase: 44±4 pA) than in VE neurons (44±31 ms; rheobase: 180±50 pA; p<0.001) (Fig. 4D). The first ISI was significantly longer in VE neurons than in LOC neurons (Fig. 4E) but did not change dramatically with the current strength in either efferent neuron type (Fig. 4C). However, a scatter plot of the first spike latency versus the first ISI revealed that, in VE neurons, these parameters were more variable whereas LOC neurons first ISI values clustered around 50 ms independently of the first spike latency (Fig. 4F). The fact that the VE and LOC neurons cluster separately from each other indicate that these cell types can be classified based on their latencies and ISIs. The slope of the depolarization leading up to the first spike (Fig. 4A) was negatively correlated (Pearson's correlation) with the first spike latency in LOC neurons (R^2^ = 0.534, p = 0.005; Fig. 4G), suggesting that these two parameters may be governed by a common mechanism. Obviously this was not the case in VE neurons that responded with a short latency, indicating a weaker or different condition underlying their initial depolarization slope.

### Voltage dependent currents in VE neurons

**Figure 5:**
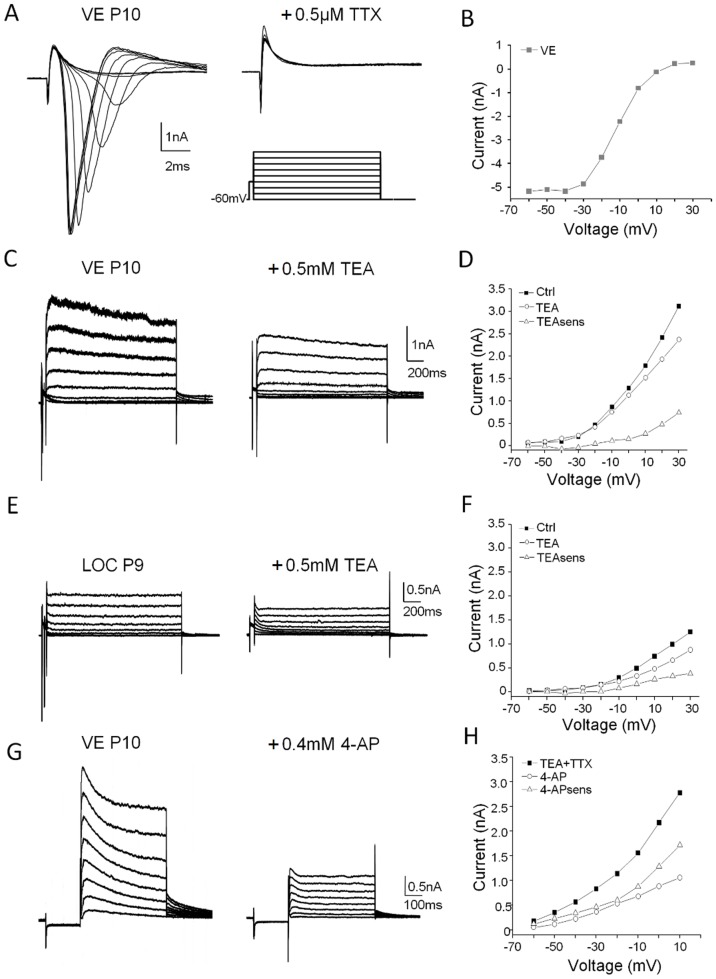
Voltage-dependent currents in the depolarizing range. Sodium currents, triggered at −30 mV in voltage clamped VE neurons were completely blocked by 0.5 µM TTX (A) and displayed systematic voltage dependency (B). Sustained outward currents, evoked by 10 mV, 1500 ms depolarizing steps from a holding potential of −60 mV (A), were partially blocked by 0.5 mM TEA in representative VE (C) and LOC (E) neurons. The sustained current was quantified at the last 100 ms of the depolarization before and after TEA application and plotted against the voltage in the VE (D) and in the LOC (F) neuron. The TEA-sensitive component was calculated by subtracting the TEA current from the control current in the respective example neuron (D and F). Transient and sustained outward currents, evoked under influence of TTX and TEA in a VE neuron by depolarizing 10 mV, 500 ms steps, preceded by a −100 mV, 50 ms hyperpolarization step to de-inactivate potential low-voltage activated currents, were partially blocked by 0.4 mM 4-AP (G). The 4-AP-sensitive component was calculated and plotted as above (H).

**Figure 6:**
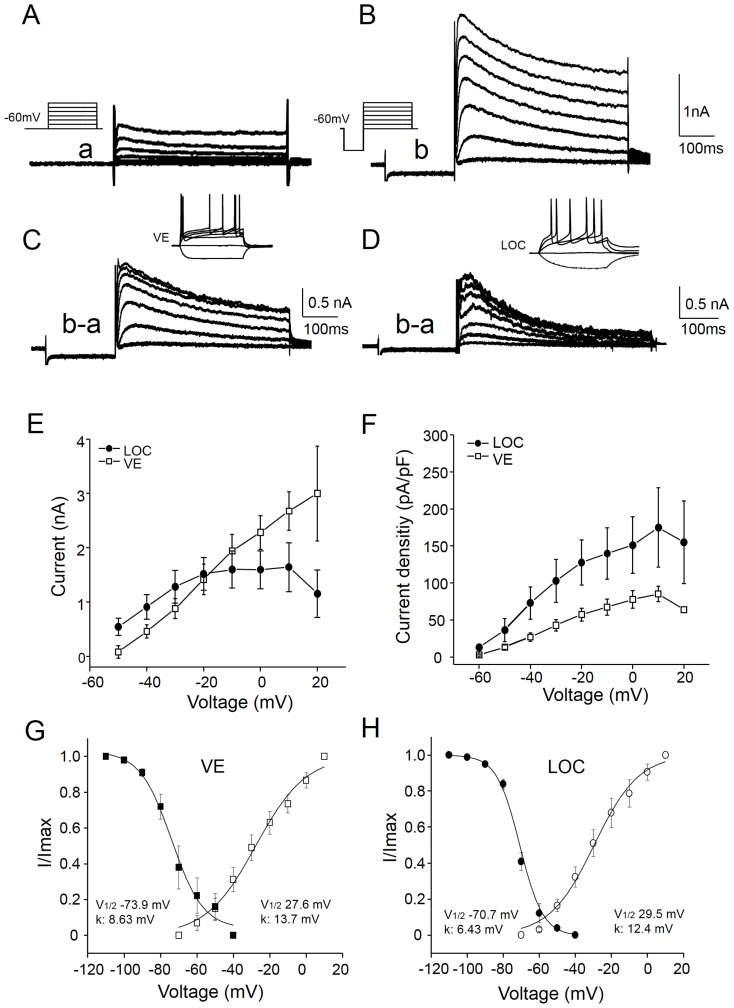
VE and LOC neurons express transient outward currents. Isolations of sustained and inactivating outward currents under influence of 0.5 µM TTX and 500 µM TEA in voltage clamped VE neurons (A–D). Two voltage clamp protocols, depolarizing the neuron in 10 mV, 500 ms, steps from a holding potential of −60 mV up to +10 mV (a), were used to trigger sustained outward currents (A). In one protocol the depolarization was preceded by a −100 mV, 50 ms hyperpolarization step to de-inactivate potential low-voltage activated currents (b). This protocol, in addition to the sustained currents, triggered large inactivating currents (B) The inactivating current component (C) was then isolated by subtracting the currents elicited in the first protocol (a) from those in the second protocol (b). The same protocols and pharmacological substances were used to isolate the transient outward current in LOC neurons (D). The insets display the identification of the neuron types in current clamp mode prior to application of the drugs. Averages of the peak transient outward currents recorded in VE (open squares, n = 6) and LOC (filled circles, n = 10) are plotted against the voltage, revealing larger currents in the VE than the LOC neurons (E). When normalizing the peak current values to the compensated capacitance for each neuron, thus obtaining the current densities, the LOC neurons express larger transient outward currents than the VE neurons throughout the voltage range tested (F). Steady state activation and inactivation curves were generated by normalizing the mean current at each voltage for the respective VE (G) and LOC (H) neurons and fitting Boltzmann functions to the data. The inactivation currents were recorded at voltages of −10 mV, which was preceded by 1.5 second voltage steps ranging from −110 to −40 mV.

Since the ion currents underlying the firing properties in the VE neurons is completely unknown, the total outward currents were recorded in eGFP-positive neurons. In order to identify possible candidate currents, a qualitative screening was first performed using a combination of voltage clamp protocols and pharmacology. To single out high and low voltage-activated outward currents, two voltage clamp protocols depolarizing the neuron in 10 mV steps from a holding potential of −60 mV were used. In the first protocol, designed to enhance high voltage-activated currents, the depolarization was preceded by a 30 mV, 50 ms depolarizing step (Fig. 5A) to inactivate potential low-voltage-activated currents. This protocol also effectively triggered fast activating and inactivating inward currents (Fig. 5A). The latter currents were confirmed to be carried by sodium channels as they were completely abolished by TTX (Fig. 5A). Both the sodium current size and kinetics were affected by the depolarizing voltage steps of the protocol, reflecting the inactivation of the sodium channels (Fig. 5B). The outward currents, elicited by the depolarizing steps were mostly sustained currents with little inactivation in VE (Fig. 5C) or purely sustained in LOC (Fig. 5E) neurons. On average, the sustained current was of similar size at 0 mV in VE (1699±118 pA; n = 3) and LOC (1630±1643 pA; n = 3) neurons but was more variable in the latter neuron type. In a second protocol, the depolarizing steps were preceded by a −100 mV, 50 ms hyperpolarizing step to enhance low-voltage-activated currents (Fig. 6B). This protocol elicited outward currents with a large inactivating component (Fig. 5H). Clearly the VE neurons are governed by at least two types of outward currents upon depolarization. It has previously been demonstrated that LOC neurons express voltage-dependent currents, which are sensitive to TEA and 4-AP [22]. Although these antagonists are not highly specific, they provide a good indication of the fraction of high (TEA) respective low (4-AP) voltage-activated currents in neurons [22]. TEA partially blocked the sustained currents in VE (Fig. 5C and D) and in LOC (Fig. 5E and F) neurons. The mean fraction of TEA-sensitive currents was 512±209 pA (n = 3) in VE and 265±211 pA (n = 3) in LOC neurons, thus accounting for 30% and 16%, respectively, of the outward currents. The remaining currents following TTX and TEA blockade in VE neurons were sensitive to 4-AP (Fig. 5G), which blocked ∼50% of the remaining outward currents in this cell (Fig. 5H). This indicates that VE neurons might be strongly governed by inactivating low voltage-activated currents upon depolarization.

### Transient outward currents have smaller impact on VE than LOC neurons

A common feature of the VE and LOC neurons is their low resting membrane potential (approx. −77 mV). A possible role for such negative membrane potential could be to keep specific voltage dependent ion channels within their working range. The LOC neurons have previously been characterized by their long delay in firing action potentials [2], a property that has been correlated to transient outward potassium currents, also known as the low-voltage activated A-type current, or I_A_ [22]. The 4-AP-sensitive inactivating outward current observed in the VE neurons would be compatible with I_A_ [32]. To further characterize the voltage dependence and kinetics of the transient depolarization-activated current, the sustained components (Fig. 6A) were subtracted from the inactivating and sustained components (Fig. 6B) using two types of voltage protocols (Fig. 6A and B). Both voltage protocols were applied under influence of TTX and TEA to improve the voltage clamp. The current subtraction revealed a transient outward current that increased in magnitude with depolarization (Fig. 6C). In identified VE neurons (Fig. 6C), the transient outward current was 2675±756 pA at 0 mV and inactivated with a time constant of 65±45 ms (n = 6). To get an estimate of the variability between neurons, the ratio between the peak current and the pseudo steady-state current was calculated at 0 mV (Fig. 6C). This inactivation index varied between 1.37 and 1.99 and the mean was 1.64±0.25; n = 6, indicating that all tested VE neurons had an inactivating outward current. For comparison, the transient outward current was also isolated in physiologically characterized LOC neurons (Fig. 6D). In LOC neurons, the amplitude was smaller than in VE neurons at 0 mV (1465±1106 pA; p = 0.033; n = 10) (Fig. 6E). However, the transient outward current displayed similar inactivation kinetics to the current recorded in VE neurons (63±57 ms at 0 mV; p = 0.94). Their inactivation index at 0 mV ranged from 1.21 to 3.64 and the mean was 2.41±2.34; p = 0.057; n = 10, indicating a larger but more variable fraction of transient outward current in LOC neurons. Interestingly, when these currents were normalized to the cell capacitance to estimate the current density, the relationship reversed. The density of the transient outward current at 0 mV was smaller in VE (78±29 pA/pF, n = 6) than in LOC (167±114 pA/pF, n = 10; p = 0.074) neurons (Fig. 6F), although values did not reach statistical significance.

The voltage dependence of the steady-state activation and inactivation was estimated by fitting a Boltzmann function to the normalized transient outward currents (I/Imax) as a function of the voltage (Fig. 6G and H). The half-activation was, respectively, −27.6 mV and −29.5 mV for VE and LOC neurons whereas the half-inactivation voltage was, respectively, −73.9 mV and −70.7 mV for VE and LOC neurons. A slope factor (the rate of inactivation) of 8.63 was estimated for VE and of 6.43 for LOC neurons. The activation and inactivation curves revealed a narrow window in which there was a small activation and incomplete inactivation of these transient outward currents in a voltage range between −70 and −40 mV (Fig. 6G and H), indicating that a fraction of these currents are probably activated around rest, and that they may be involved in the regulation of sub-threshold fluctuations of the voltage in both VE and LOC neurons.

### VE and LOC neurons express Kv4.3 and Kv4.2 potassium channel subunits

The voltage dependence and the transient nature of the TEA isolated outward currents in VE and LOC neurons is highly compatible with the low voltage-activated A-type currents mediated by the Kv4 family of ion channels [4]. This ion current is mediated via the Kv4 channel family, which includes three pore-forming α-subunits: Kv4.1, Kv4.2 and Kv4.3; of which Kv4.2 and Kv4.3 are specific for the brainstem [21], [33], [61]. In order to investigate if Kv4 subunits are expressed in VE neurons and, in that case which subtype, immunolabeling against ChAT, identifying the VE and LOC neurons, was combined with Kv4.2 and Kv4.3 antibodies in wild type animals.

**Figure 7:**
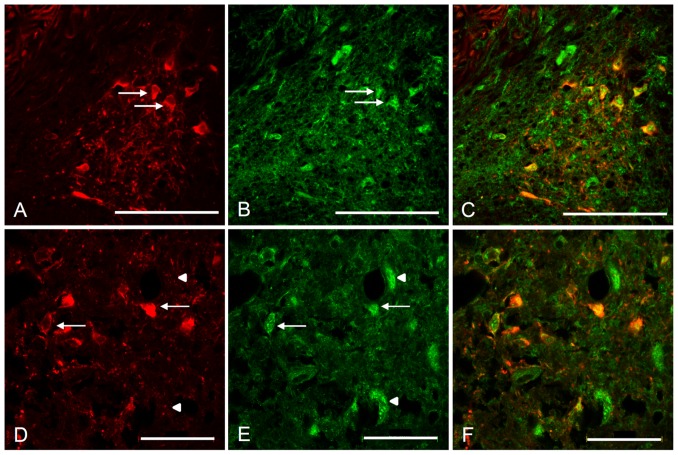
VE and LOC neurons express Kv4.3 subunits in the rat. Confocal laser scanning microscopy images of adult rat immunofluorescently labeled against ChAT (red) and Kv4.3 (green). The ChAT labeled VE (A–C) and the LOC (D–E) neurons are double labeled with Kv4.3, indicating that they are expressing the protein of the Kv4.3 α-subunit. Arrows indicate double labeled efferent neurons and arrowheads point to cells labeled against Kv4.3, but lacking ChAT immunolabeling, most probably corresponding to principal neurons in the lateral superior olive (D–E). Scale bars: (A–C) 100 µm, (D–F) 50 µm.

As the Kv4 antibodies used in the present study have been confirmed to produce specific immunolabeling of Kv4.2 and Kv4.3 in rat cochlear nucleus [57], the expression of these channel subunits was initially investigated in the rat. The immuonolabeling displayed robust expression of Kv4.3 in both VE (Fig. 7A–C) and LOC (Fig. 7D–F) neurons, as evident from the overlap of ChAT and Kv4.3 immunoreactivity in high resolution confocal images (Fig. 7C and F) The immunoreaction was observed both in the cell body and in the proximal dendrites for both neuronal types (Fig. 7C and F).

**Figure 8:**
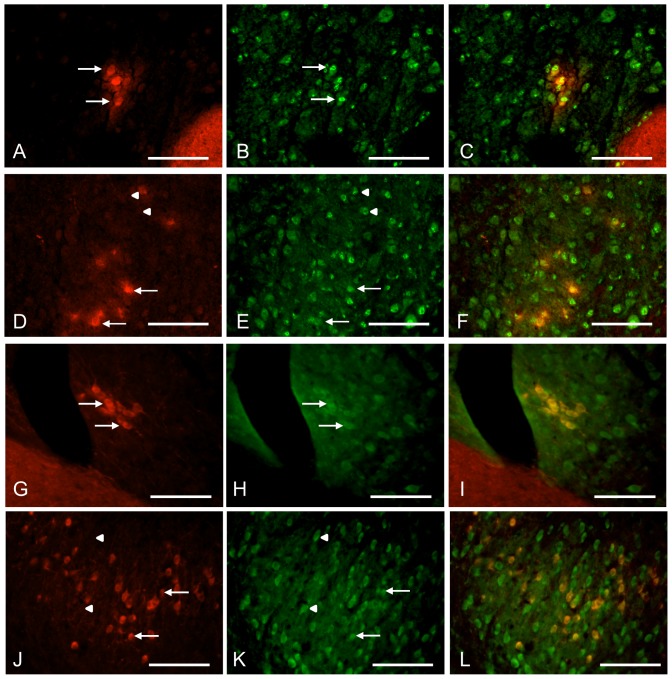
VE and LOC neurons express Kv4.3 and Kv 4.2 subunits in the mouse. Adult mouse immunofluorescently labeled against ChAT (red) and Kv4.2 or Kv4.3 α-subunits (green) using shock-frozen tissue. The ChAT labeling overlaps with the Kv4.3 labeling in the mouse VE neurons (A–C). Likewise, both the ChAT-positive LOC efferent cells (arrows) and the principal cells of the lateral superior olive (arrowheads) show Kv4.3 expression in the mouse (D–F). In a similar fashion, both the VE neurons (G–I) and the LOC efferent cells (arrows) and the principal cells of the lateral superior olive (arrowheads) (J–L) are immunolabeled against Kv4.2, which indicate that the Kv4 channels are hetero-meric in these auditory brainstem nuclei. Scale bars: 100 µm.

In order to verify the expression-pattern of Kv4.3 in the mouse brainstem, immunohistochemistry was performed on fixed mouse brain tissue with the same procedure used for the rat tissue. However, this protocol failed to produce any specific Kv4.3 staining in the mouse. In accordance with another study of Kv4-immunolabeling in mice [33], the tissue preparation was switched from trans-cardial perfusion to a protocol using shock-freezing of the brain tissue. Presumably, this method better preserved antigenicity or opened up the cell membrane for better antibody-targeting of intracellular epitopes. Using this preparation of the brain tissue, Kv4.3 immunolabeling could be confirmed in both VE (Fig. 8A–C) and LOC (Fig. 8D–F) neurons in the mouse.

Since the Kv4.2 and Kv4.3 α-subunits often hetero-tetramerize to form the native ion channel [4], one would also suspect some level of Kv4.2 expression in the same areas as for the Kv4.3. A strong Kv4.2 expression was indeed observed in mouse VE (Fig. 8G–I) and LOC neurons (Fig. 8J–L). In rat, however, the Kv4.2 expression could not be confirmed. Taken together, these results strongly suggest that Kv4 family potassium channel is underlying the transient outward A-type currents, previously documented in the LOC efferent neurons [22] and here demonstrated in the VE neurons. More specifically, Kv4 channels in the VE and LOC neurons consist of both Kv4.3 and Kv4.2 α-subunits, presumably forming hetero-meric channel pores.

## Discussion

### Differential expression of eGFP-ChAT in VE and olivocochlear neurons

The absence in the literature of intracellular recordings from the brainstem VE neurons is related to the difficulty of morphologically defining this small neuronal group without a specific marker. We reasoned that the presently used BAC transgenic mouse, in which eGFP is knocked into the first ChAT-coding exon (exon 3) and that preserves most of the regulatory elements [66], would have higher chances of selective expression of eGFP in all cholinergic cells in the brain. Indeed, eGFP was expressed in the vestibular efferents and this was instrumental for the targeted patch-clamp recordings performed in this study. Given the highly specific location in a small confined area dorsolateral of the genu of the facial nerve in mammals [11], [24], [46] and the co-localization with ChAT-immunoreactivity, we feel confident that we recorded from VE neurons. Somewhat surprisingly, however, eGFP was neither expressed in LOC nor in MOC neurons, both of which neuronal types are cholinergic [9], [11], [69]. This discrepancy may be related to the complex regulation of the ChAT gene, which include both enhancer and suppressor elements making it notoriously difficult to robustly express markers like GFP e.g. [41], [50]. Another complicating factor is the alternative splicing of ChAT mRNA [37], [48], which has been reported in various types of cholinergic neurons [67]. It is, thus, possible that the olivocochlear neurons express an alternative splice variant of ChAT, which leads to a loss of eGFP expression. Recordings from LOC neurons were possible in this mouse model as they were readily identified based on their physiological properties in the lateral superior olive without GFP-labeling [2], [62]. However, although individual LOC neurons displayed similar membrane and firing properties, both their transient and sustained outward currents displayed more inter-cell variability than VE neurons. We can, thus, not exclude that we have recorded from subgroups of LOC neurons [55], [36].

### Comparison between VE neurons and the olivocochlear efferents

In the present study, the VE neurons were, for the first time, physiologically investigated and compared to other inner ear-projecting neurons. The discussion that follows is accordingly aimed to interpret and speculate on the functional role of the VE neurons, based on their intrinsic electrical properties compared to LOC neurons.

The most obvious similarity between VE and LOC neurons is their negative resting membrane potential. However, most other basic membrane properties and the spike shape differed between VE and LOC neurons in age-matched animals (Table 2), indicating different functional roles of the two inner-ear-projecting neuron types. Also their firing response patterns differed in fundamental ways. Upon depolarization, VE neurons displayed clear onset spiking followed by sparse, regular firing with long ISIs whereas LOC neurons typically fired tonically with a long first spike latency. The question is: what currents contribute to these distinct properties in VE and LOC neurons? Voltage-gated K^+^ channels play fundamental roles in controlling neuronal excitability and firing patterns in most neurons. LOC neurons are known to express the low-voltage activated potassium current I_A_ [22], which is known to contribute to a long first spike latency, or so called ‘delayed firing’ [63]. The A current, carried by the Kv4 channels [4], is a rapidly activating and inactivating K^+^ current [32]. I_A_ is characteristically active at the resting membrane potential, usually demonstrated as a ‘window-current’ in the −70 to −50 mV range [33], [63], [70]. The large transient outward currents, with instantaneous activation and rapid inactivation upon depolarization, being active at resting membrane voltages in VE neurons, are highly compatible with I_A_. Immunohistochemistry demonstrated robust expression of both Kv4.3 and Kv4.2 subunits in VE neurons, which further supports that I_A_ is an important player for regulating the excitability of VE neurons, presumably carrying the A currents as hetero-merized Kv4.3 and Kv4.2 channel pores. LOC neurons, confirmed to express I_A_ [22] with similar voltage sensitivity and kinetics to the VE neurons, also expressed Kv4.3 and Kv4.2 channels. However, as VE neurons were at least twice the size of the LOC neurons, based on capacitance measurements, the A current density was reduced in the VE neurons. As a consequence I_A_ has much less impact on the initial spiking response in VE neurons compared to in LOC neurons, in which the spiking is shunted. Thus, both VE and LOC neurons use their negative membrane potentials to keep the same subset of low-voltage activated Kv4 channels within their operating range but the respective ion channel density determines the power of I_A_ in the two neuronal types. It is quite possible that I_A_ also contributes differentially to the integration of synaptic inputs [63], regulation of excitability [19] and back-propagating action potentials [13] in VE and LOC neurons, respectively.

Another hallmark of VE neurons is their slow firing of action potentials, regulated by their pronounced AHP. Even if transient outward currents have been shown to be important for action potential repolarization and regulation of the inter-spike interval [15], [70], it is tempting to speculate that VE neurons express additional K-currents to the Kv4-mediated currents that are not blocked by TEA. This speculation is further supported by the lower inactivation index in VE neurons compared to in LOC neurons (Fig. 6C). Other potassium channel subfamilies that are insensitive to TEA that could come in question are the Kv1 [63] or the Kv7 [8] channels, both of which carry low-voltage-activated and non-inactivating currents. A combination of the fast activating Kv1 and Kv4-mediated currents, could hypothetically promote high-pass filtering at the onset of a depolarization in VE neurons, shunting weak or slow depolarizations in favour of coincident strong synaptic inputs for action potential generation [65]. The Kv7-mediated current, which is slowly activating, is known to control the intrinsic excitability and the spike rate by contributing to the AHP in hippocampal neurons [64], [68]. Likewise, a Kv7-mediated current might be a candidate to contribute to the sparse, regular firing with pronounced AHP in VE neurons observed during prolonged depolarizations.

### How do the intrinsic properties of VE neurons relate to their functional role in vivo?

It has been shown in mammals [25], [45] and in toadfish [5] that efferent activation predominantly excites vestibular afferents. Specifically, VE activation causes an increase in background discharge activity of the primary afferents in combination with reduced response gain, i.e. reduced response amplitudes to sensory stimulation. The strictly excitatory effect of vestibular efference stands in stark contrast to the inhibitory effects observed during efferent activation in the cochlea, which is inhibitory and serves to dampen the cochlear amplification and to protect the auditory system from over-stimulation [16].

It has been hypothesized that the efferent vestibular system functions to modulate the response magnitude of the afferents during the large accelerations accompanying volitional motion [25]. Moreover, in toadfish, increased VE neuron activity has also been associated with arousal and predation [26], further building on a hypothesis that the main function of the vestibular efferent system is to reduce vestibular-evoked responses triggered by self-generated motion in favour of biologically-relevant sensations. However, this notion has been refuted by a seminal experiment performed in alert macaques in which VE-modulation of the vestibular primary afferent response did not differ between voluntary or passively applied head movements [17]. Thus, based on the above mentioned literature, it seems more likely that vestibular efferents may raise the overall activity in the nerve to bring the vestibular afferents into their optimal working range (i.e. the linear range of their input-output function); [25]. It has also been suggested that the vestibular efferents may also increase the bandwidth of the system [12] by lowering the impedance in the vestibular hair cells through opening of SK-channels [29]. The question is: how could the intrinsic properties of VE neurons tie in with the functions of the vestibular system?

One of the main functions of the vestibular system is to signal abrupt head movements during rotational acceleration by the semi-circular canal organs [47]. This would require a highly phasic response pattern, which abates as the stimulation wears off. The estimated cupular time constant in mice is 3.7 seconds [35], which is a much slower time scale than the *in vitro* experiments conducted herein. However, it is possible that the robust onset response of VE neurons to depolarizing stimuli reflects a capacity to boost activity in the vestibular afferents triggered by the fast (10–100 ms) component of cupular activation whereas the delayed, non-adapting response pattern of the VE neurons is reflecting an augmentation of the slow (3–20 s) component [35]. In line with this idea, vestibular efferent-evoked increases in activity in semi-circular canal afferents (especially the ones with irregular discharge patterns) that consisted of distinct fast and slow response components, has been recorded in alert and anesthetized monkeys [59], [25], in chinchilla [43] and cat [45]. Interestingly, the feed-back excitation from the VE system does not seem to play a role in the compensation of a unilateral vestibular loss [31], [58]. This finding seems to rule out a bilateral activity-equalizing role of VE neurons similar to what has been ascribed to LOC neurons, the function of which seem important for balancing the auditory nerve activity between the two sides [18].

An alternative interpretation of the VE response pattern is that their rather regular responses with low gain (i.e. small change in response to increasing current stimulation) would be suitable to provide tonic feed-back to the macular organs that mediate gravity-induced vestibular tonus of the body [47]. This would explain why the maculae and peripheral zones of the cristae receive ample innervation from VE neurons [39], [40], [53]. Either functional role, i.e. phasic modification of semi-circular canal afferent discharge rate or tonic feed-back to gravito-sensing organs, are perhaps not mutually exclusive.

The second important question that arises is: what drives the VE neurons *in vivo*? The above reasoning of a ‘vestibular gain control’ strongly implies that VE neurons may be part of a feed-back loop from the vestibular afferents, as has been suggested in a model by Plotnik et al. [52]. VE neurons indeed get ample innervation from the vestibular primary afferents [28], [34], [38] and a mono-synaptic reflex loop in the brainstem would be consistent with the fact that the VE efferents can be driven up to approximately 100 Hz before they saturate [6]. Another input may come from the second order Type I neurons [60] in the vestibular nuclei, as demonstrated by retrograde tracing of VE neurons in the rat [14]. Notably, VE neurons also get a multitude of inputs from autonomic centers, such as hypothalamic nuclei, reticular formation, solitary tract and dorsal vagus nuclei [46]. It is conceivable that VE neurons are subject to neuromodulation in stressful situations [49], which may enhance their excitability and increase their sensitivity to vestibular stimulation. If this is the case, they might provide a powerful link between the well-known relation between emotional stress and dizziness [30] and motion sickness [42].

### Conclusions

It seems fundamental to be able to control the inner ear organs with the brain and yet the functional role of vestibular efference to the balance system remains elusive. This study provides the first direct characterization of the brainstem neurons that project to the inner ear vestibular end-organs where they exert control over primary afferents and hair cells. The intrinsic physiological properties of VE neurons might be compatible with their highly diverse and unspecific projections throughout vestibular sensory epithelia, providing feed-back amplification of abrupt and strong phasic signals from the semi-circular canals and of tonic signals from the gravito-sensitive macular organs.
